# Integrating MRI-based radiomics and clinicopathological features for preoperative prognostication of early-stage cervical adenocarcinoma patients: in comparison to deep learning approach

**DOI:** 10.1186/s40644-024-00747-y

**Published:** 2024-08-01

**Authors:** Haifeng Qiu, Min Wang, Shiwei Wang, Xiao Li, Dian Wang, Yiwei Qin, Yongqing Xu, Xiaoru Yin, Marcus Hacker, Shaoli Han, Xiang Li

**Affiliations:** 1 Department of Gynecology, the First Affiliated Hospital of Zhengzhou University, No.1, east Jian she Road, Zhengzhou, 450000 Henan Province China; 2 Evomics Medical Technology Co., Ltd, Shanghai, China; 3 Department of Obstetrics and Gynecology, Qilu Hospital of Shandong University, Jinan, Shandong Province China; 4 Division of Nuclear Medicine, Department of Biomedical Imaging and Image-Guided Therapy, Vienna General Hospital, Medical University of Vienna, Vienna, Austria; 5 Department of Nuclear Medicine, Beijing Tuberculosis and Thoracic Tumor Research Institute, Beijing Chest Hospital, Capital Medical University, Beijing, China

**Keywords:** Cervical adenocarcinoma, Radiomics, Machine learning, Deep learning, T2-weighted MRI image, Disease-free survival

## Abstract

**Objectives:**

The roles of magnetic resonance imaging (MRI) -based radiomics approach and deep learning approach in cervical adenocarcinoma (AC) have not been explored. Herein, we aim to develop prognosis-predictive models based on MRI-radiomics and clinical features for AC patients.

**Methods:**

Clinical and pathological information from one hundred and ninety-seven patients with cervical AC was collected and analyzed. For each patient, 107 radiomics features were extracted from T2-weighted MRI images. Feature selection was performed using Spearman correlation and random forest (RF) algorithms, and predictive models were built using support vector machine (SVM) technique. Deep learning models were also trained with T2-weighted MRI images and clinicopathological features through Convolutional Neural Network (CNN). Kaplan-Meier curve was analyzed using significant features. In addition, information from another group of 56 AC patients was used for the independent validation.

**Results:**

A total of 107 radiomics features and 6 clinicopathological features (age, FIGO stage, differentiation, invasion depth, lymphovascular space invasion (LVSI), and lymph node metastasis (LNM) were included in the analysis. When predicting the 3-year, 4-year, and 5-year DFS, the model trained solely on radiomics features achieved AUC values of 0.659 (95%CI: 0.620–0.716), 0.791 (95%CI: 0.603–0.922), and 0.853 (95%CI: 0.745–0.912), respectively. However, the combined model, incorporating both radiomics and clinicopathological features, outperformed the radiomics model with AUC values of 0.934 (95%CI: 0.885–0.981), 0.937 (95%CI: 0.867–0.995), and 0.916 (95%CI: 0.857–0.970), respectively. For deep learning models, the MRI-based models achieved an AUC of 0.857, 0.777 and 0.828 for 3-year DFS, 4-year DFS and 5-year DFS prediction, respectively. And the combined deep learning models got a improved performance, the AUCs were 0.903. 0.862 and 0.969. In the independent test set, the combined model achieved an AUC of 0.873, 0.858 and 0.914 for 3-year DFS, 4-year DFS and 5-year DFS prediction, respectively.

**Conclusions:**

We demonstrated the prognostic value of integrating MRI-based radiomics and clinicopathological features in cervical adenocarcinoma. Both radiomics and deep learning models showed improved predictive performance when combined with clinical data, emphasizing the importance of a multimodal approach in patient management.

**Supplementary Information:**

The online version contains supplementary material available at 10.1186/s40644-024-00747-y.

## Introduction

Cervical cancer (CC) remains a leading cause of cancer-related deaths in women worldwide surpassing other gynecological tumors [1]. The burden of CC is particularly pronounced in developing countries, resulting in significant socio-economic implications [2, 3]. In 2022, China witnessed over 111,820 new cases and 61,579 deaths attributed to CC, underscoring the urgency for effective diagnostic and treatment strategies [4]. Among the various types of CC, adenocarcinoma (AC), constituting 20% of cases, is notably aggressive and linked to worse outcomes compared to squamous cell carcinoma (SCC) [5, 6]. AC and its precancerous stages often originate in the endocervix, making them difficult to detect through standard HPV + PAP smear screenings due to their inconspicuous nature and tendency for skip lesions, thereby necessitating reliance on imaging modalities like MRI for diagnosis [4, 6]. Key clinical prognostic factors for AC include tumor stage, size, para-uterine invasion, and metastasis, with AC showing higher recurrence rates than SCC despite available treatments [7]. Despite the availability of comprehensive treatments, AC is associated with a higher recurrence rate than SCC [8, 9]. For early-stage CC patients, the conventional approach often involves radical hysterectomy combined with pelvic lymph node dissection [10]. The ability to accurately predict prognostic markers before surgery could significantly enhance treatment planning and prognostication [5, 11]. Magnetic resonance imaging (MRI) serves as a crucial noninvasive tool for CC diagnosis and staging [12, 13], offering detailed insights into tumor morphology and extent within the pelvis, including potential bladder and rectal invasion, and predicting responses to neoadjuvant chemotherapy [14, 15]. Furthermore, radiomics models derived from MRI data can predict lymph node metastasis (LNM) and lymphovascular space invasion (LVSI), critical factors in determining post-operative care and patient outcomes [16, 17].

The deep learning (DL) model as a newly emerging model, allows the automatic discovery of the representations with the use of fully connected layers in the network and can analyze the nonlinear correlations that are more common in the real world [18]. Several deep learning models have been reported to be effective in the diagnosis, treatment stratification, and prognostic prediction for CC [19–21]. Notably, DL models have demonstrated efficacy in various studies, including precise identification of deep stromal invasion in AC and cervical adenosquamous carcinoma, and accurate segmentation of gross tumor volume in CC patients [22, 23]. Additionally, DL models have shown potential in predicting outcomes for non-surgical CC patients based on pathological image analysis [24].

Despite AC representing a smaller fraction of the overall CC population, the exploration of MRI-based radiomics and deep learning in AC remains limited. This study aims to investigate the performance of MRI-based radiomics and deep learning models in the context of AC, addressing a critical gap in the current research landscape.

## Methods

### Patients

**Fig. 1:**
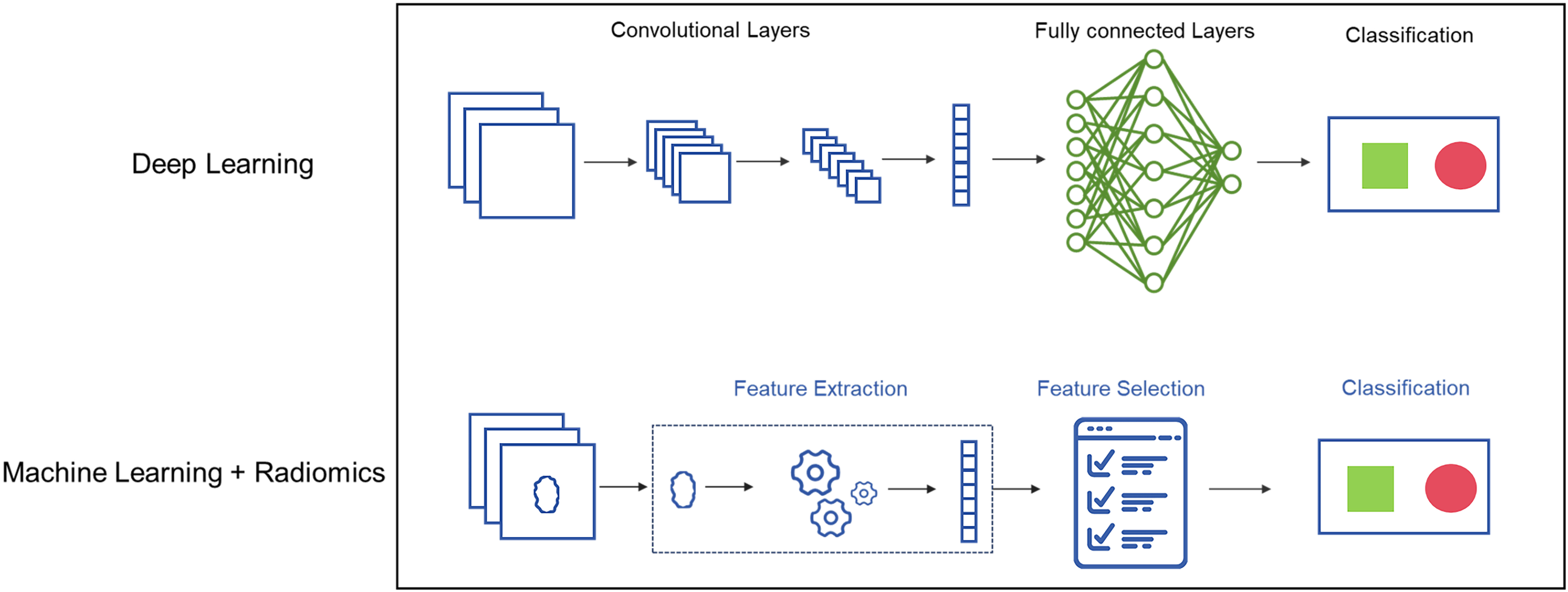
The pipeline of this study. Radiomics-based predictive model construction: Firstly, manual segmentation of ROI on MRI. Then feature extraction and feature selection were performed using Spearman correlation and random forest. Model training and validation was performed through SVM. MRI-based deep learning predictive construction: MRI with or without clinicopathological features were included for model training through deep learning network. ROI, Region of Interest; RF, Random Forest; SVM, Support Vector Machine

This retrospective study was approved by the ethical committee at the First Affiliated Hospital of Zhengzhou University. A total of 216 AC patients who took a surgery in our hospital were recruited for this study, from December 1st, 2013 to October 31st, 2019. After the preliminary treatments, follow up was performed every 2–3 months in first and second year, then every 6 months in third and fourth year, and once a year in the fifth year and later. During this period, 19 patients were lost before the third year and thus excluded for the final analysis. The inclusion criteria were as follows: (1) patients with stage Ia1-IIA2 AC (usual-type) and accepted (radical) hysterectomy ± lymphadenectomy was qualified for recruitment; (2) MRI was performed within one week before the surgery; (3) no previous treatment was given prior to the MRI examination. The exclusion criteria were: (1) before MRI, the biopsy for pathological diagnosis was permitted, but conization or LEEP (loop electrosurgical excision procedure) was not qualified; (2) poor image quality; (3) rare histological subtypes (like gastric-type, neuroendocrine carcinoma, clear cell, and serous); (4) patients who accepted NACT (neoadjuvant chemotherapy treatment) was ruled out to avoid the impacts on final pathology results. The diagnosis was re-confirmed by an experienced pathologist. Clinical-pathological parameters were extracted from medical records. Tumor stages were determined according to the FIGO 2018 criteria. If there was no suspicious lymph node metastasis (indicated by MRI, CT, or other imaging tests), the patients with stage IA1 tumor (invasion depth < 3.0 mm, determined by conization or LEEP) did not undergo the systematic lymphadenectomy. All the other patients accepted the systematic lymphadenectomy (both pelvic and paraaortic lymphadenectomy) according to the NCCN guidelines, version 2023. For the independent test cohort, another group of 56 AC patients were recruited from Qilu Hospital of Shandong University (the time of surgery ranged from June 1st, 2014 to Dec 31st, 2020). The pipeline of this study is illustrated in Figure 1.

### MRI acquisition protocol

All scans were performed on a Siemens Syra 3.0T MRI scanner (Siemens, Germany), 18 channel surface phased array coil. The T2-weighted (T2W) sequence which are routinely performed along with T1-weighted (T1W) was used for the analysis. The details of the scan parameters were shown in Table 1.

**Table 1:** The details of the scan parameters for T2-weighted (T2W) sequence acquisition

Parameters	Axial T2WI
TR (msec)	3000
TE (msec)	116
Slice thickness (mm)	4
FOV	180 mm×180 mm
Matrix	384 × 269

Enhancement scans were performed by a rapid (< 10 s) bolus injection of gadopentetate dimeglumine (Gd-DTPA) via the elbow vein with a high-pressure syringe at a dose of 0.2 mmol/kg and a rate of 2–3 ml/s. One phase of plain scanning was conducted before injection, and 23 phases of uninterrupted repeat scanning were performed after injection.

### Regions of interest segmentation

3D Slicer software Version 4.13 was used to delineate the whole cervix uteri as volume of interest (VOI) on the axial orientation T2-weighted images manually by a radiologist of three years of experience, and confirmed by a senior radiologist of 5 years of experience [25].

### Radiomics feature extraction and feature engineering

For each patient, a total 107 radiomics features was extracted using the “PyRadiomics” package implemented in Python [26]. The radiomics features included: (1) 14 shape-based features; (2) 18 first-order features; (3) 24 GLCM features; (4) 16 GLRLM features; (5) 16 GLSZM features; (6) 14 GLDM features; (7) 5 NGTDM features.

To address collinearity among the radiomics features, we performed an initial reduction step using Spearman correlation analysis. Features with a correlation coefficient greater or equal to 0.8 were considered redundant and removed from further analysis. In addition to radiomics features, we collected 6 clinicopathological features for each patient: age, FIGO stage, differentiation degree, invasion depth, LVSI, and LNM. The reduced radiomics features were then combined with the clinicopathological features for final feature selection. Random forest was also applied to select the features that were important to the prognosis. Based on random forest, the contribution value of each feature on each tree in a random forest was estimated. Specifically, the contribution value was calculated based on the Gini index.

### Machine learning predictive models

After feature selection, support vector machine (SVM) was used to build prediction models. Cross-validation scheme was applied with 80% training and 20% validation ratios across 5 folds. To correct the sample imbalance of different labels, ‘class_weight’ was set to ‘balanced’ mode in the model of each fold. Before model training, RandomizedSearchCV was used to perform hyper-parameters fine-tuning from the specified parameter space, and the best parameters was selected based on area under the curve (AUC). It should be noted that the random forest feature selected and hyperparameter fine-tuning were conducted in the inner loop cross validation of the training set, and the validation set was only used to evaluate the performance of the model. In addition to AUC, other indicators such as accuracy, sensitivity, specificity, PPV, NPV are also used to evaluate model performance. Predictive models of 3-year DFS, 4-year DFS and 5-year DFS were trained using radiomics features alone as well radiomics features combined with clinicopathological features.

### Kaplan-Meier analysis

According to the random forest feature importance, we further selected the four most important features and performed Kaplan-Meier analysis to validate the prognostic value of these features. In which, the continuous variables were divided into high-value and low-value groups based on the optimal truncation value generated by R package survminer (Version 0.4.9), while category variables were directly used to draw Kaplan-Meier curve.

### Deep learning prognostic predictive model

We first converted the spacing of the MRI images of T2 sequence to the median of the data spacing of this batch: (0.5468, 0.5468, 6). Since the position of the cervix in MRI images is relatively fixed, and there are many redundant areas, we applied fixed size center crop to the data after the unified spacing: (224,224,18). Finally, Z-Score normalization was performed on the trimmed data.

The dataset was randomly divided into training and validation cohorts by 8:2. Our model adopted the self-developed convolutional neural network structure and the batch size is set to 8 during training. The details of the model structure and training were described in supplementary files.

### Statistical analysis

The area under the receiver operating characteristics curve (ROC AUC), sensitivity, specificity, positive predictive value, negative predictive value and accuracy were used to assess the discrimination performance of the machine learning models. SVM and ROC curve visualization was performed by using the Python (v3.8) package scikit-learn (v1.2.0) and matplotlib (v 3.5.1). Kaplan-Meier curve was analyzed using the R package survival (v3.2-13). *P*-value less than 0.05 was considered statistically significant.

## Results

### Patients characteristics

The baseline characteristics of 197 AC (usual-type) patients were provided in Table 2. The median age at diagnosis was 45.5 years-old (ranged from 26 to 72). The median DFS was 51 months (ranged from 5 to 115 months). According to the FIGO 2018 staging system, 133 women (67.5%) exhibited stage I (6 IA1, 10 IA2, 70 IB1, 32 IB2, 15 IB3) and 27 (13.7%) had disease stages II tumors (17 IIA1, 8 IIA2 and 1 IIB). The other 37 cases (18.8%) belong to stage III (1 IIIB, 35 IIIC1p, and 1 IIIC2p). As for the histology, 28 (14.2%), 123 (62.4%), and 46 (23.4%) cases were high, medium, and low differentiation. As for the tumor size, 96 (48.7%), 61 (31.0%), and 40 (20.3%) tumors are divided into ≤ 2 cm, 2–4 cm, and > 4 cm groups. According to the invasion depth, 58 (29.4%), 96 (48.7%), and 43 (21.8%) patients presented with < 1/3, 1/3 − 2/3, and > 2/3 depth of invasion. 30.5% (*n* = 60) and 18.3% (*n* = 36) cases were positive with LVSI and LNM.

**Table 2:** Baseline clinical parameters of AC patients. LVSI, lymphovascular space invasion; LNM, lymph node metastasis

Variables	*n* (%)
Median age (range)	45.5 (26–72) years-old
Grade	
High	28 (14.2)
Medium	123 (62.4)
Low	46 (23.4)
Tumor size	
≤ 2 cm	96 (48.7)
2–4 cm	61 (31.0)
> 4 cm	40 (20.3)
FIGO stage	
IA1	6 (3.0)
IA2	10 (5.1)
IB1	70 (35.5)
IB2	32 (16.2)
IB3	15 (7.6)
IIA1	17 (8.6)
IIA2	8 (4.1)
IIB	2 (1.0)
IIIB	1 (0.5)
IIIC1p	35 (17.8)
IIIC2p	1 (0.5)
LVSI	
Negative	137 (69.5)
Positive	60 (30.5)
Depth of invasion	
< 1/3	58 (29.4)
1/3 − 2/3	96 (48.7)
> 2/3	43 (21.8)
LNM	
Negative	161 (81.7)
Positive	36 (18.3)

As shown in Table 3, the median age at diagnosis was 49.5 years-old. In the independent test group (ranged from 26 to 72). The median DFS was 37 months (ranged from 4 to 74 months). 41 women (73.2%) exhibited stage I (1 IA1, 25 IB1, 9 IB2, 6 IB3) and 2 (3.6%) had disease stages IIA1 tumors. The other 13 cases (23.2%) belong to stage III (12 IIIC1p, and 1 IIIC2p). As for the histology, 10 (17.9%), 26 (46.4%), and 20 (35.7%) cases were high, medium, and low differentiation. As for the tumor size, 28 (50.0%), 16 (28.6%), and 12 (21.4%) tumors are divided into ≤ 2 cm, 2–4 cm, and > 4 cm groups. According to the invasion depth, 20 (35.7%), 13 (23.2%), and 20 (35.7%) patients presented with < 1/3, 1/3 − 2/3, and > 2/3 depth of invasion. 23.2% (*n* = 13) and 23.2% (*n* = 13) cases were positive with LVSI and LNM (Fig. 1).

**Table 3:** Baseline clinical parameters of AC patients in the independent test group. LVSI, lymphovascular space invasion; LNM, lymph node metastasis

Variables	*n* (%)
Median age (range)	49.5 (26–72) years-old
Grade	
High	10 (17.9)
Medium	26 (46.4)
Low	20 (35.7)
Tumor size	
≤ 2 cm	28 (50.0)
2–4 cm	16 (28.6)
> 4 cm	12 (21.4)
FIGO stage	
IA1	1 (1.8)
IA2	0 (0.0)
IB1	25 (44.6)
IB2	9 (16.1)
IB3	6 (10.7)
IIA1	2 (3.6)
IIA2	0 (0.0)
IIIC1p	12 (21.4)
IIIC2p	1 (1.8)
LVSI	
Negative	43 (76.8)
Positive	13 (23.2)
Depth of invasion	
< 1/3	20 (35.7)
1/3 − 2/3	13 (23.2)
> 2/3	20 (35.7)
LNM	
Negative	43 (76.8)
Positive	13 (23.2)

### Feature selection and radiomics-based machine learning model performances

**Fig. 2:**
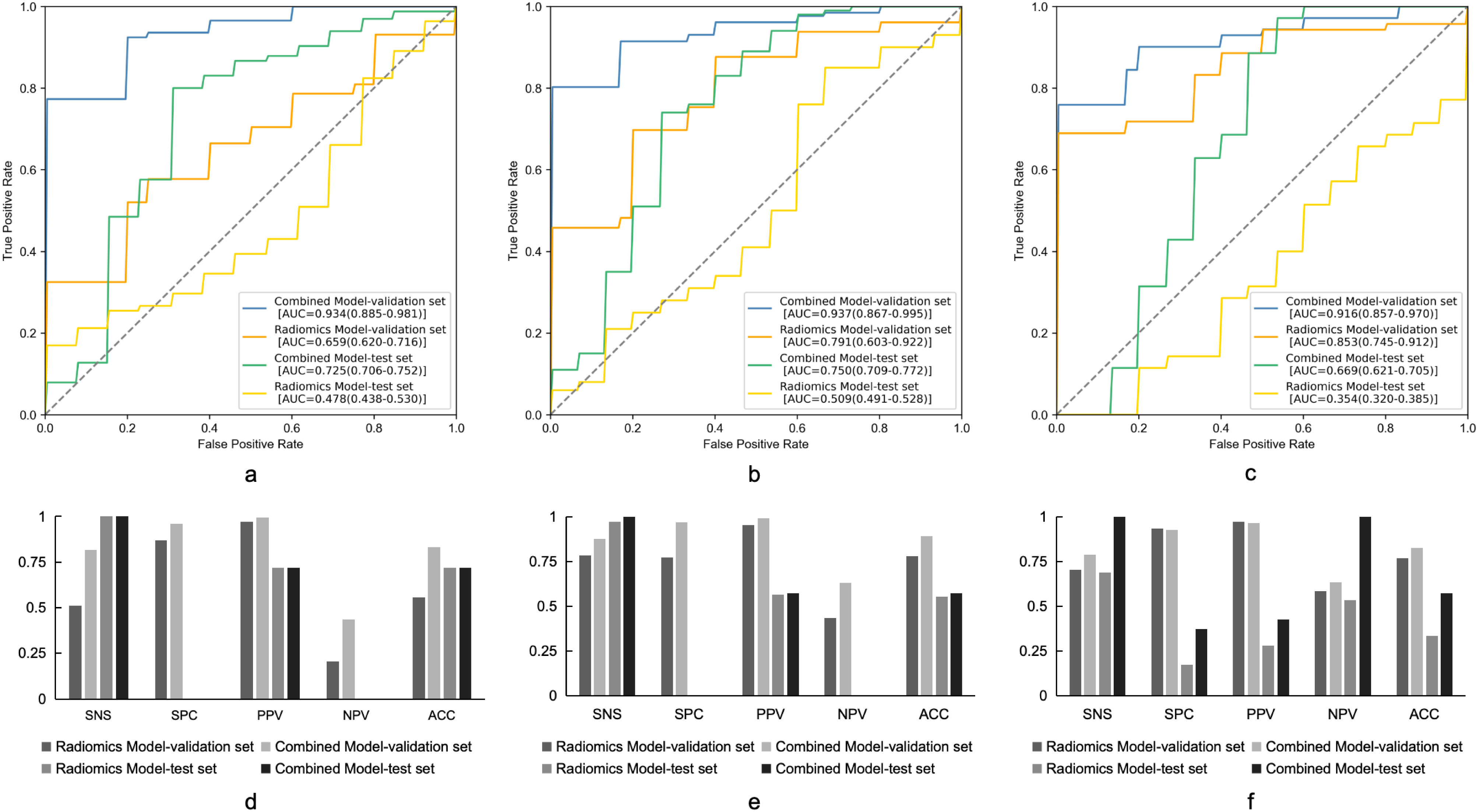
ROC curves of radiomics-based predictive models of 3-year DFS (**a**), 4-year DFS (**b**) and 5-year DFS (**c**). The other performance matric of the predictive models of 3-year DFS (**d**), 4-year DFS (**e**) and 5-year DFS (**f**) were also shown. SNS, sensitivity; SPC, specificity; PPV, positive predictive value; NPV, negative predictive value; ACC, accuracy. DFS, disease-free survival

The fifteen features with the highest weight were remained for the model construction. For 3-year DFS prediction, the model trained with radiomics features alone achieved an AUC of 0.659 (95%CI: 0.620–0.716). The combined model got a better performance than the radiomics model (AUC 0.934, 95% CI: 0.885–0.981). The combined model also had a better performance in sensitivity, specificity, positive predictive value, negative predictive value and accuracy (Fig. 2a and d).

For 4-year DFS prediction, the radiomics model yielded an AUC of 0.791 in the validation set (95%CI: 0.603–0.922). And the combined model showed performance improvement (AUC 0.937, 95%CI: 0.867–0.995). The other performance indicators were also better in the combined model (Fig. 2b and e).

We got a similar result in the prediction of 5-year DFS, the combined model demonstrated an AUC of 0.916 (95%CI: 0.857–0.970), while the radiomics model got an AUC of 0.853 (95%CI: 0.745–0.912). The other performance indicators were also better in the combined model (Fig. 2c, g and f).

### Kaplan-Meier analysis of selected features

**Fig. 3:**
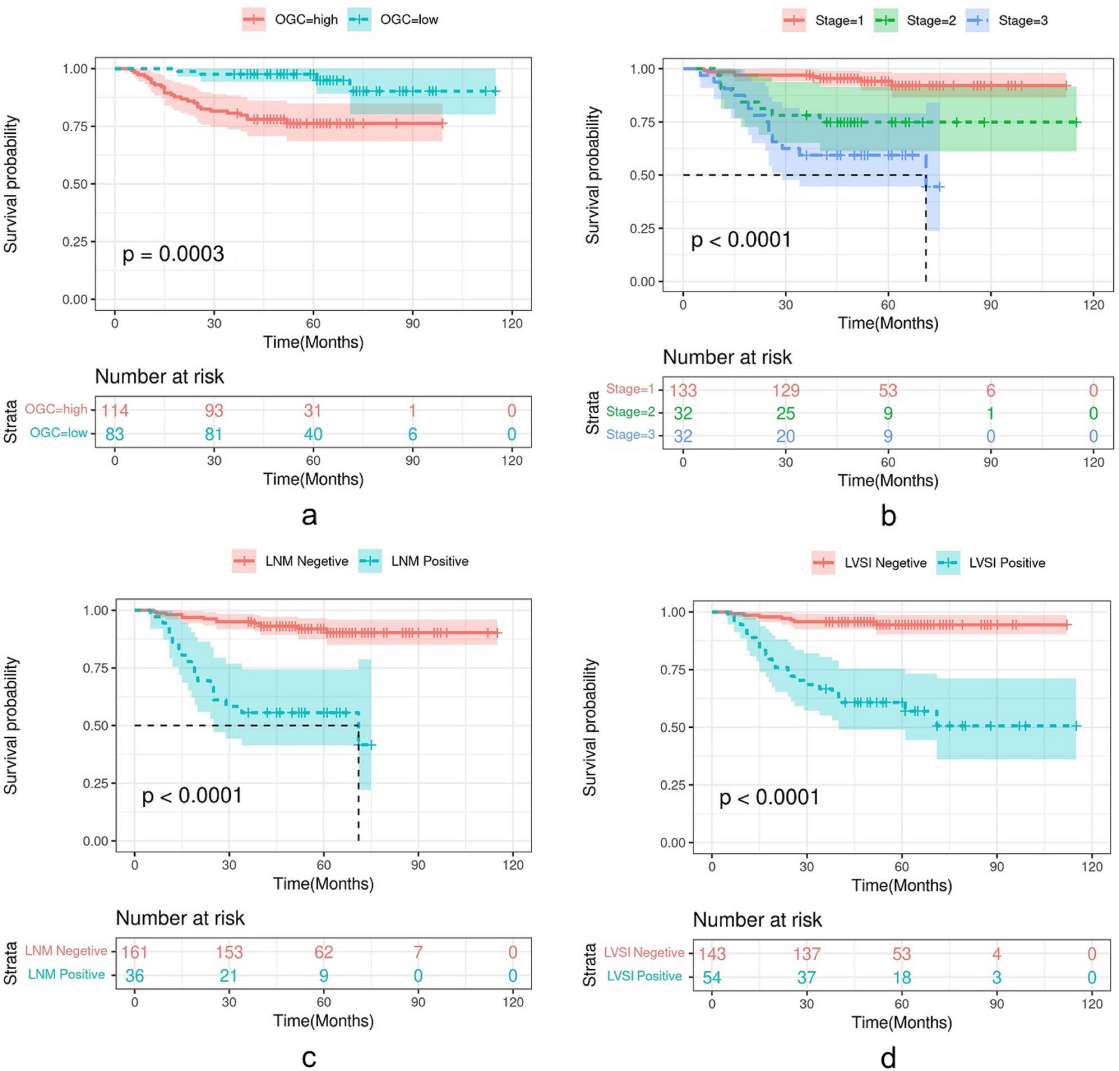
Kaplan-Meier analysis of selected features. **A**) Kaplan-Meier curves of the radiomics feature OGC. **B-D** represent Kaplan-Meier curves of the FIGO stage, LVSI and LNM. Shadows represent 95% CI. LVSI, lymph-vascular space invasion; LNM, lymph node metastasis; OGC, original_glcm_Correlation. CI, confidence interval

The Kaplan-Meier analysis was used for the validation of the prognostic value of the selective radiomics and clinicopathological features. One radiomics features original_glcm_Correlation (OGC) and three clinicopathological features including FIGO stage, LNM and LVSI were selected to perform Kaplan-Meier analysis. As shown in Fig. 3, higher OGC (*p* = 0.0003), stage 3 (*p* < 0.0001), LNM (*p* < 0.0001) and LVSI (*p* < 0.0001) positive were all significantly associated with worse DFS. Especially, FIGO stage and LNM had more obvious impact for prognostic value, which patients had only 50% survival probability for FIGO stage 3 group or LNM positive group undergo 30 months. While OGC and LVSI didn’t have so important impact for prognostic value.

**Fig. 4:**
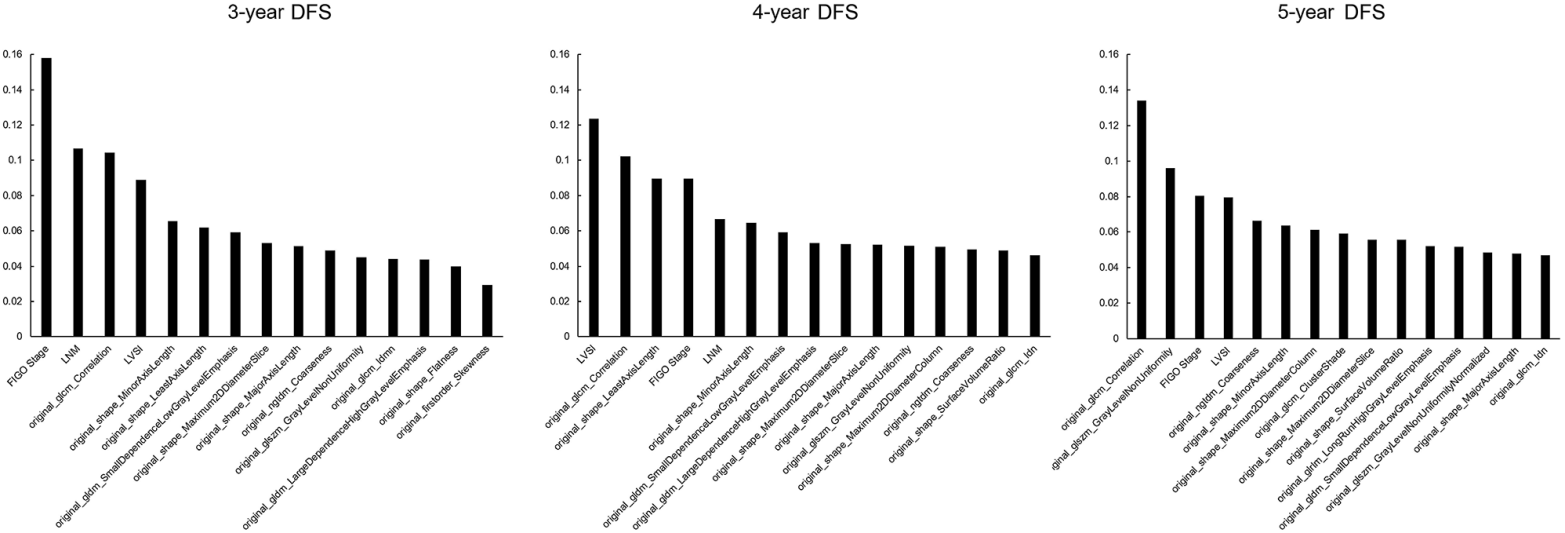
Feature Importance of the combined model of 3-year DFS, 4-year DFS and 5-year DFS by Built-in Random Forest Importance

**Fig. 5:**
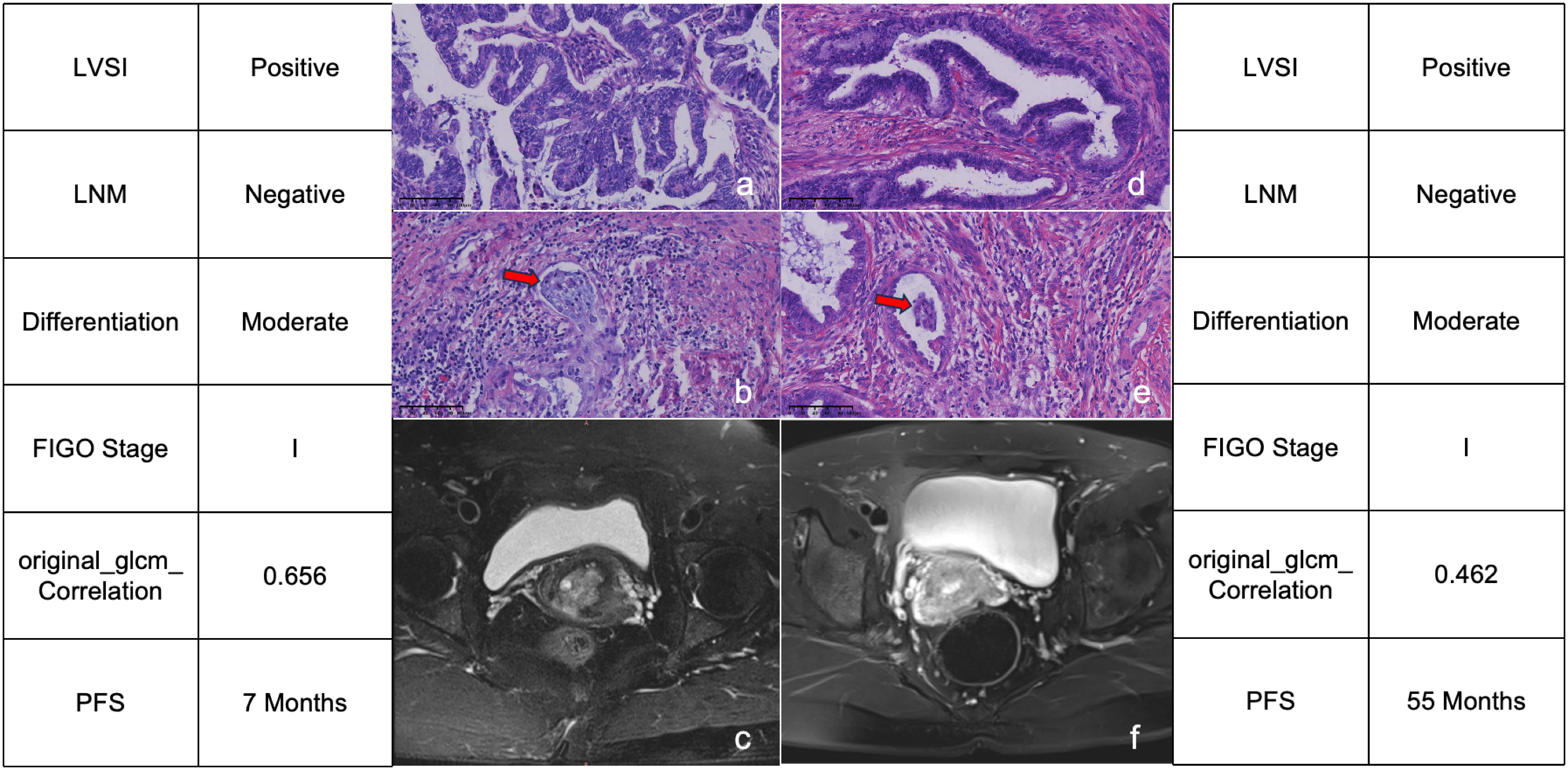
Representative H&E staining and MRI scans of 2 patients who presented poor prognosis or better prognosis. H&E staining (**a**), H&E staining marked by LVSI (**b**) and MRI scan (**c**) for the patient with poor prognosis. H&E staining (**d**), H&E staining marked by LVSI (**e**) and MRI scan (**f**) for the patient with better prognosis

The feature importance of the models was calculated through RF method. For 3-year DFS model, the first three important features were FIGO stage, LNM and original_glcm_Correlation. While for 4-year DFS model and 5-year DFS model, the three most important features included two radiomics features and one clinicopathological feature. Especially for 5-year DFS model, the first two important features were both radiomics features (original_glcm_Correlation and original_glszm_GrayLevelNon Uniformity) (Fig. 4). Representative MRI scans of two patients with poor prognosis or better prognosis were illustrated, the corresponding values of the most related features were recorded (Fig. 5).

### MRI-based deep learning predictive model performances

**Fig. 6:**
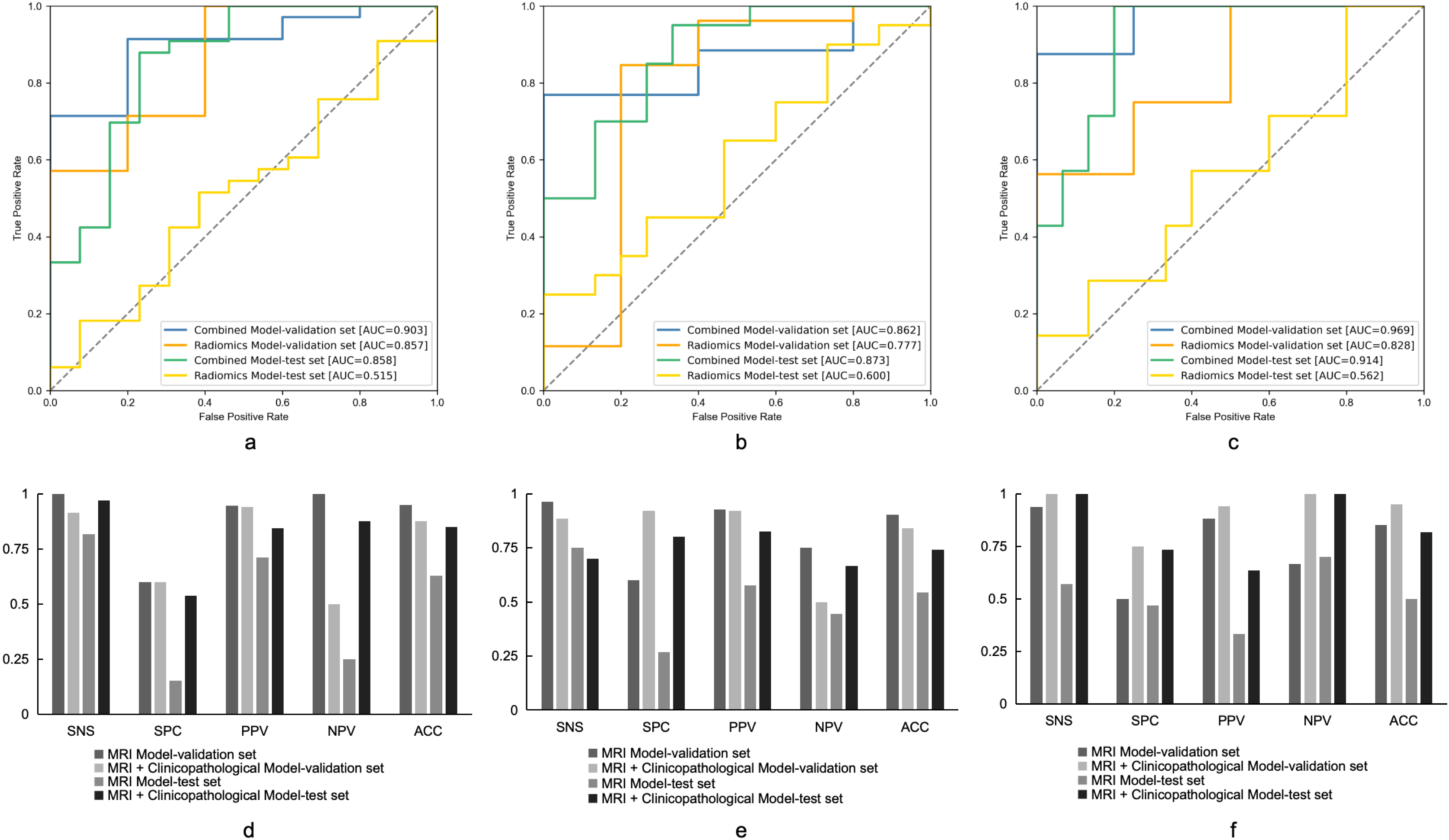
ROC curves of deep learning predictive models of 3-year DFS (**a**), 4-year DFS (**b**) and 5-year DFS (**c**). The other performance matric of the predictive models of 3-year DFS (**d**), 4-year DFS (**e**) and 5-year DFS (**f**) were also shown. SNS, sensitivity; SPC, specificity; PPV, positive predictive value; NPV, negative predictive value; ACC, accuracy. DFS, disease-free survival

We also trained predictive models with T2W images and clinicopathological features through deep learning approach. MRI-based deep learning models achieved an AUC of 0.857, 0.777 and 0.828 for 3-year DFS, 4-year DFS and 5-year DFS prediction, respectively in the validation cohort. While for the combined deep learning models, they got a better performance than the MRI-based deep learning models. The AUCs of the 3-year DFS, 4-year DFS and 5-year DFS prediction models were 0.903, 0.862 and 0.969, respectively (Fig. 6).

### Models performances confirmed by the independent test cohort

A total of 56 AC patients were recruited for the independent test analysis. The model performances for both of the radiomics-based machine learning model and deep learning predictive model were also shown in Figs. 2 and 6. Under the radiomics-based machine learning models, the combined model achieved an AUC of 0.725 (95%CI: 0.706–0.752), 0.750 (95%CI: 0.709–0.772) and 0.669 (95%CI: 0.621–0.705) for 3-year DFS, 4-year DFS and 5-year DFS prediction, respectively in the independent test set (Fig. 2). The combined model got a better performance than the radiomics model, which was consistent with the validation results. Under the deep learning predictive models, the combined model achieved an AUC of 0.873, 0.858 and 0.914 for 3-year DFS, 4-year DFS and 5-year DFS prediction, respectively in the independent test set (Fig. 6), which was also consistent with the validation results on a better performance than the MRI-based deep learning models.

## Discussion

According to the world health organization (WHO) classification system, the usual-type AC accounts for about 80% of all ACs, following with other rare histologic subtypes such as gastric-type, clear cell, mesonephric, endometrioid, and others [27]. In this study, we developed and validated prognostic models for early-stage cervical adenocarcinoma (AC) patients by integrating MRI-based radiomics and clinicopathological features. Our key findings indicate that the combined model, which incorporates both radiomics and clinicopathological features, outperformed models based solely on radiomics features in predicting 3-year, 4-year, and 5-year disease-free survival (DFS). Specifically, the combined model achieved higher AUC values, indicating better predictive accuracy and robustness. Additionally, deep learning models that incorporated clinicopathological features demonstrated enhanced performance compared to those using MRI images alone. These results underscore the importance of combining clinical data with advanced imaging techniques to improve prognostic predictions in cervical AC patients.

Cervical AC is usually more aggressive, exhibiting a greater propensity for local and distant metastasis, reduced responsiveness to radiotherapy and chemotherapy, and an unfavorable prognosis [28, 29]. While conventional MRI has demonstrated efficacy in predicting risk factors and prognosis in CC, the emerging field of radiomics has garnered attention due to its ability to objectively and accurately extract quantitative features from MRI images, including density, contour, volume, and texture [30]. In this study, we present, for the first time, the prognostic value of MRI in a cohort of patients specifically diagnosed with the usual-type cervical AC through radiomics-based machine learning method and deep learning method, respectively. Our results reveal that the radiomics model achieved AUC values of 0.659, 0.791, and 0.853 for predicting 3-year, 4-year, and 5-year DFS, respectively. Notably, while the 3-year DFS prediction was relatively modest, it demonstrated a gradual increase over time, suggesting the potential of radiomics in predicting long-term disease-free survival. OGC appeared importance in all prognostic models, it is a first-order GLCM feature reflecting on the information content of the linear dependency of gray level values in the image. A higher correlation implies a greater linear relationship between the gray levels of pixel pairs, often used to assess the heterogeneity or complexity of the texture in medical images. Thus, this OGC can be critical in analyzing textures in radiology images, helping in the treatment planning, and prognosis of diseases by highlighting patterns not always visible to the human eye. For the MRI-based deep learning models, the AUC values were 0.857, 0.777 and 0.828 for 3-year, 4-year and 5-year DFS, respectively. The deep learning model presented a better performance than the radiomics-based model for 3-year DFS prediction.

Earlier investigations have also explored the predictive capacity of MRI radiomics in cervical cancer. In a prior study encompassing 191 cases of early SCC, the incorporation of age, FIGO stage, LVSI, and other indicators into the model construction revealed a significantly superior predictive value of the radiomics model for DFS compared to traditional clinical models [31]. Similarly, a separate study involving 378 stage I-II cervical cancer patients, including a subset of 33 AC cases, demonstrated the extraction of textural features from T2-weighted and ADC data, which, when combined with clinical pathological parameters using stepwise logistic regression, yielded an impressive AUC of 0.916 for predicting tumor recurrence [32]. Furthermore, a study utilizing the least absolute shrinkage and selection operator (LASSO) regression and Cox proportional hazard model, incorporating 248 CC patients, including 33 AC cases, reaffirmed the superior predictive value of MRI radiomics for DFS compared to traditional clinical pathological parameters [33]. Collectively, these findings underscore the promising predictive value of MRI radiomics in early CC and provide objective evidence to inform clinical decision-making. However, the majority of current researches in the realm of MRI radiomics in cervical cancer primarily concentrates on SCC, leaving the value of radiomics in AC inadequately explored. Given the significant disparities and inherent heterogeneity between AC and SCC, it is imperative to investigate the potential of MRI radiomics specifically in cervical AC.

Commonly employed prognostic indicators in clinical practice encompass FIGO stage, tumor size, depth of cervical stromal invasion, LVSI, and LNM [34]. Drawing upon parameters from the Surveillance, Epidemiology, and End Results (SEER) database, Ni et al. [35] identified histological grade, T stage, N stage, M stage, tumor volume, and surgical intervention as independent prognostic indicators for cervical AC. Similarly, Zhou et al. [36] conducted a retrospective analysis of stage I-IIB cervical AC, revealing LNM and age as independent prognostic indicators for overall survival, while tumor volume and LNM were independent prognostic indicators for recurrence-free survival. In a cohort comprising 305 cases of cervical AC, researchers validated 5-year overall survival rates of 80%, 37%, and 11% for stages I, II, and III, respectively [37]. In this present study, we incorporated clinical parameters alongside cervical AC radiomics to establish a comprehensive model, resulting in significantly improved AUC values. Furthermore, clinical parameters were also added into the deep learning models and also presented improved performances. This enhancement in predictive performance underscores the potential benefits of amalgamating objective and accurate MRI images with clinical indicators, particularly for midterm and long-term prognosis. However, the need for confirmation through large-scale, multicenter, randomized controlled studies cannot be understated.

Several limitations must be acknowledged in our study. All patients included in our analysis presented with early-stage tumors and underwent extensive hysterectomy accompanied by lymph node dissection. Postoperative adjuvant treatments were administered according to authoritative guidelines such as those provided by the National Comprehensive Cancer Network (NCCN). Additionally, all patients exhibited the usual type of cervical adenocarcinoma, ensuring consistency in case selection and minimizing significant biases, thereby enhancing the reliability and reproducibility of our results. However, in accordance with the FIGO 2018 staging system, some patients were pathologically confirmed to have lymph node metastasis, potentially resulting in stage upgrading and introducing confounding factors. Despite these limitations, we believe that our study, being the sole and most expansive radiomics investigation focused on usual-type cervical adenocarcinoma, contributes novel tools for clinical diagnosis, treatment selection, and prognosis prediction. Another limitation of this study is that the sample size is insufficient for deep learning model construction, and more samples need to be added to optimize the model. Moreover, the retrospective design, the single-center nature of our study, and the reliance on a specific patient population might limit the applicability of our findings to broader contexts. Finally, Only T2 sequence was used in this study could limited the performance of established model, the decision to omit other sequence, e.g. DWI was made due to its inherent variability in interpretation, which could potentially compromise the uniformity of our dataset. Thus, our study primarily emphasized T2w MRI sequences to ensure consistency across the entire cohort. Moreover, T2w MRI sequences are more ubiquitously available for AC imaging and are deemed more appropriate for facilitating cross-center validation efforts. The potential confounding effects of clinical and pathological parameters and how our analyses help mitigate these influences, thereby providing a more accurate prognostic model.

## Conclusion

In our study, we demonstrated the prognostic power of integrating MRI-based radiomics and clinicopathological features for evaluating usual-type cervical adenocarcinoma. Both radiomics and deep learning models showed enhanced predictive accuracy when combined with clinical data. These findings advocate for the use of a multimodal approach, integrating radiomics with clinical and pathological information, to improve prognostication and guide clinical decision-making in cervical adenocarcinoma.

### Electronic supplementary material

Below is the link to the electronic supplementary material.

Supplementary Material 1

## Data Availability

The datasets used during the current study are available from the corresponding author on reasonable request.

## Abbreviations

AC: Adenocarcinoma
ACC: Accuracy
AUC: Area under the curve
CC: Cervical cancer
CI: Confidence interval
CNN: Convolutional neural network
DFS: Disease-free survival
Gd-DTPA: Gadopentetate dimeglumine
LASSO: Least absolute shrinkage and selection operator
LEEP: Loop electrosurgical excision procedure
LNM: Lymph node metastasis
LVSI: Lymphovascular space invasion
MRI: Magnetic resonance imaging
NACT: New adjuvant chemotherapy treatment
NCCN: National comprehensive cancer network
NPV: Negative predictive value
OGC: Original_glcm_correlation
PPV: Positive predictive value
RF: Random forest
ROC: Receiver operating characteristics
SCC: Squamous cell carcinoma
SEER: Surveillance, epidemiology, and end results
SNS: Sensitivity
SPC: Specificity
SVM: Support vector machine
VOI: Volume of interest
