# Supplementary material for: Integrating MRI-based radiomics and clinicopathological features for preoperative prognostication of early-stage cervical adenocarcinoma patients: in comparison to deep learning approach
Source: Cancer Imaging. 2024 Aug 1;24:101. doi: 10.1186/s40644-024-00747-y (PMC11292990; doi:10.1186/s40644-024-00747-y)
Supplement: Supplementary file 1 — Supplementary Material 1 [file 40644_2024_747_MOESM1_ESM.docx]

**The details of the deep learning models structure and training process**

Our model adopted the self-developed convolutional neural network structure as shown in the Figure S1, and the batch size is set to 8 during training. When the batch size was set as 5, on the patch embedding, the number of feature map channels on the patch embedding would increase from 18 to 128. As the size of the feature map was small after tailoring and patch embedding, we would not downsample when extracting features in backbone, but repeat 8 residual structure blocks, each block was convolution by kernel_size=3. Composed of Batch Normalization(BN) and Rectified Linear Unit (ReLU), the feature maps in the backbone were all of the same size (44,44,128).

In order to enrich features and improve model performance, we performed global average pooling and global maximum pooling on feature graphs in parallel before the full connection layer. In an experiment combining clinical parameters and image data to train the models, we input 6 clinical parameters together with pooling results into the fully connected layer.

All the experimental data were divided randomly in the ratio of 8:2. The epoch for training was set to 400. The loss function used in training was Binary Cross-Entropy Loss. The optimizer of the model adopted the Stochastic Gradient Descent (SGD) with initial learning rate of 0.05 and weight decay of 1e-3. During training, the learning rate would gradually decrease with the epoch number. The model was trained on a separate GPU (NVIDIA RTX 3090 32 GB), and it took about 4 hours to complete all the models.


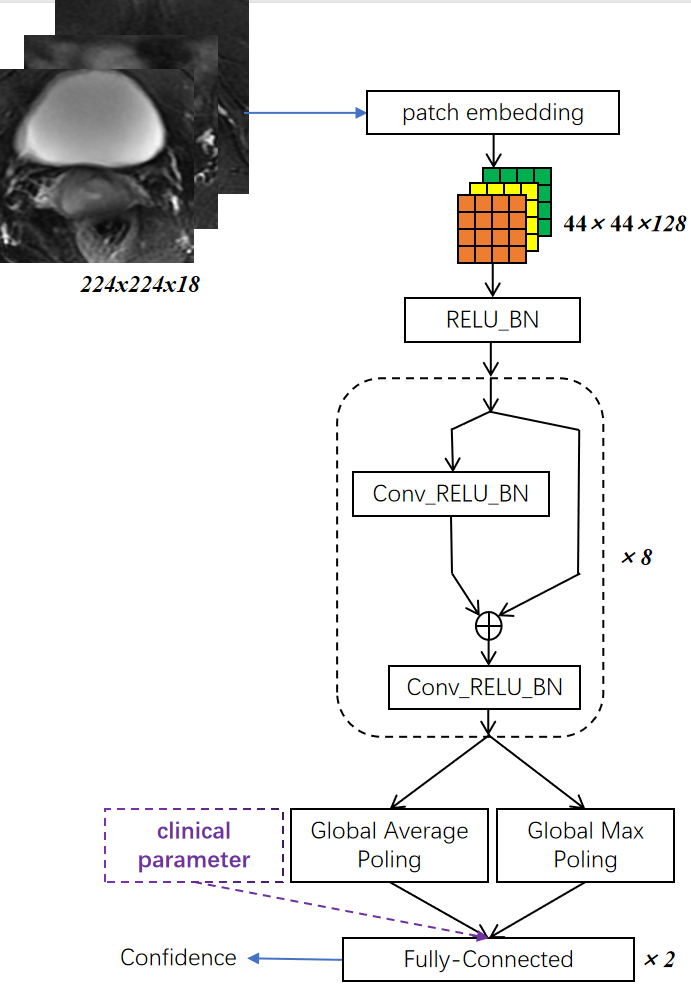


**Figure S1** The structure of convolutional neural network.
